# Application of Nanoparticles for Targeting G Protein-Coupled Receptors

**DOI:** 10.3390/ijms19072006

**Published:** 2018-07-10

**Authors:** Xin Ma, Yunfang Xiong, Leo Tsz On Lee

**Affiliations:** Centre of Reproduction Development and Aging, Faculty of Health Sciences, University of Macau, Taipa, Macau, China; florazgnd@gmail.com (X.M.); yb77617@connect.umac.mo (Y.X.)

**Keywords:** G protein-coupled receptor (GPCR), cancer, nanoparticles (NPs), dendrimers, quantum dots (QDs), gold nanoparticles (AuNPs), magnetic nanoparticles (MNPs)

## Abstract

Nanoparticles (NPs) have attracted unequivocal attention in recent years due to their potential applications in therapeutics, bio-imaging and material sciences. For drug delivery, NP-based carrier systems offer several advantages over conventional methods. When conjugated with ligands and drugs (or other therapeutic molecules), administrated NPs are able to deliver cargo to targeted sites through ligand-receptor recognition. Such targeted delivery is especially important in cancer therapy. Through this targeted cancer nanotherapy, cancer cells are killed with higher specificity, while the healthy cells are spared. Furthermore, NP drug delivery leads to improved drug load, enhanced drug solubility and stability, and controlled drug release. G protein-coupled receptors (GPCRs) are a superfamily of cell transmembrane receptors. They regulate a plethora of physiological processes through ligand-receptor-binding-induced signaling transduction. With recent evidence unveiling their roles in cancer, GPCR agonists and antagonists have quickly become new targets in cancer therapy. This review focuses on the application of some notable nanomaterials, such as dendrimers, quantum dots, gold nanoparticles, and magnetic nanoparticles, in GPCR-related cancers.

## 1. GPCR Activation and GPCRs in Cancer

G protein-coupled receptors (GPCRs) are membrane receptors that make up the largest family of cell surface receptors of the human genome [1]. GPCRs are also called seven-transmembrane (7TM) receptors because of the common structural motif shared by their family members. Based on sequence homology and phylogenetic data, human GPCRs are classified into six groups: Class A comprises of rhodopsin receptors; class B has two subclasses—secretin receptors (B1) and adhesion receptors (B2); class C comprises of glutamate receptors; class F comprises of frizzled receptors, and class T comprises of taste two receptors [2]. GPCRs can convert foreign stimuli, ranging from particles as small as protons to large proteins, into intracellular signals through different mechanisms [3,4]. In the classical model of receptor activation, GPCR signaling is mediated by guanine nucleotide-binding proteins (G proteins) upon ligand-receptor binding. G proteins associated with GPCRs are heterotrimeric and composed of three subunits: α-, β- and γ-. In the basal state, Gα is anchored to the inner surface of cell membranes and bound to GPCR, guanosine diphosphate (GDP), Gβ and Gγ. When a ligand activates GPCR, an exchange of GDP to guanosine triphosphate (GTP) takes place. This event results in a monomeric GTP bound form of Gα, a Gβγ dimer, and the dissociation of the Gα-GTP from the receptor. The freed Gα-GTP monomers and Gβγ dimers can regulate effector enzymes, such as adenylyl cyclases, phospholipases, and ion channels, which in turn induces a series of downstream signaling cascades [5].

When GPCR is activated, it undergoes conformational changes. The G protein-coupled receptor kinases (GRKs) recognize activated receptors and phosphorylate GPCRs on specific sites, while β-arrestins are recruited for receptor desensitization (dissociation of G protein and GPCR). In contrast to the classical view, a “biased activation” mode was proposed, upon uncovering the evidence of GPCR activation via β-arrestin. Arrestins were originally recognized for their roles in GPCR desensitization. In the biased activation mode, GPCRs recruit either the G protein-dependent pathways, or the β-arrestin-dependent pathways, where β-arrestin mediates a range of GPCR signaling transductions. The molecular mechanism of biased activation is not fully understood; however, it is speculated that both GPCR conformational stabilization and downstream pathways are different between G protein-biased ligand activation and arrestin-biased ligand activation [4,6]. In addition to the two GPCR activation modes mentioned above, a “transactivation” mode has also been proposed. The traditional transactivation refers to the GPCR ligands activating receptor tyrosine kinases (RTKs), such as GPCR agonists activating epidermal growth factor receptors (EGFRs) and platelet-derived growth factor receptors (PDGFRs). The underlying mechanisms of this activation involve the generation of RTK ligand precursors after GPCR activation [7], or the formation of a GPCR-RTK receptor signaling complex, where activated G protein subunits can be used by RTKs and trigger a RTK downstream signaling cascade [8,9]. On the other hand, it is established that the crosstalk between the two receptor families is bidirectional. The mechanisms of GPCR transactivation are similar to those of RTK transactivation, which involve the synthesis of cognate GPCR ligand or GPCR-RTK complex formation [7]. The expression level of the Gα subunits may influence the biased signal of GPCR and even the transactivation of RTKs [10]. In the naïve state, the level of Gα expression affects not only Gα signalling but also the co-expressed receptor within different membrane domains. These evidences proposed a unique model to control for RTK activation via targeting GPCR complexes. This crosstalk between RTK and GPCR signalling systems regulates several cellular processes; the dysfunctional signal integration between the two receptors may sometimes result in a variety of disease states, such as cardiovascular and renal disorders, obesity, metabolic syndrome, type diabetes mellitus, cancer, etc. The understanding of GPCR activation is fundamental for targeted cancer therapy. However, due to the complexity of the biased activation and GPCR transactivation, this review only covers GPCR targeted therapy based on classical ligand-receptor binding.

GPCRs regulate nearly all the physiological processes, playing important roles in multiple systems, such as the immune system, cardiovascular system, neuron system, reproductive system and sensory system. GPCR dysfunction causes a variety of diseases, including diabetes, hypertension, Alzheimer’s disease, anxiety, asthma and cancer [11]. It is now understood that mutations in GPCR genes and irregularities in GPCR signaling pathways can cause carcinogenesis and cancer progression [12,13]. Notable GPCRs linked to cancer include lysophosphatidic acid receptors (LPARs), chemokine receptors, gastrin-releasing peptide receptors (GRPRs) and angiotensin II (ANGII) receptors. Lysophosphatidic acid (LPA) is a glycerophospholipid, and widely regarded as a cancer inducer. Its physiological role includes cell proliferation, cell migration, DNA synthesis and cytokine production. Alterations in LPA circulation profiles and LPAR expression levels can lead to cancer genesis and progression, which are demonstrated in ovarian, thyroid, colon, breast and pancreatic cancer [14,15,16]. For example, the study by Yu et al. revealed that a majority of ovarian cancer cells aberrantly overexpress LPAR2 and LPAR3 [16]. The roles LPARs play in promoting cancer survival and growth, and inhibiting migration and invasion of cancer cells, are well documented. LPRAs stimulate multiple downstream signaling pathways such as the MAPK/ERK pathway, P13K-Akt pathway and Rho-signaling pathway. Several LPAR antagonists are currently being investigated for cancer treatment. For instance, drugs based on isoxazole and thiazole are being used to treat pancreatic cancer metastasis [12]. Chemokines are chemotactic cytokines. They function in cell–cell interactions and cell migration. High levels of chemokine production can induce aberrant chemokine receptor activation and promote tumor growth and metastasis. Among the fifty identified chemokines, CXCL1-3, CXCL8, CCL2 and CCL5 are thought to be linked to tumor progression. Chemokine receptor 4 (CXCR4) is believed to be a driver of tumor metastasis and is overexpressed in ovarian cancer cells, breast cancer cells and acute lymphoblastic leukemia [1,13,17]. Various drugs targeting CXCR4 activation and downstream signaling have been developed. For example, the CXCR4 antagonist plerixafor (AMD-3100) inhibits angiogenesis within cancer xenografts in mice and is being tested in clinical trials. In another example, treatment with PIM kinase inhibitors efficiently blocks CXCR4-induced migration by receptor dephosphorylation [18]. GRP, also called mammalian bombesin (BBN), functions as a neuropeptide and a growth factor. Both GRPs and GRPRs are found overexpressed in many cancers, including gastric, lung, prostate, and breast cancer. In a study conducted on 1432 primary breast cancer samples, GRPRs were found to be overexpressed in over 75% of the cases. Interestingly, when GRPRs were overexpressed in breast tumors, a high level of GRPR expression was also detected in metastatic lymph nodes in about 95% of the cases [19]. Activated GRPRs can induce the MAPK/ERK signaling pathway and Rho-signaling pathway [20]. The high binding affinity of GPRP towards cancer cells makes it a promising candidate for targeting cancer cells. GRPR antibody 2A11 is currently in clinical use for lung cancer treatment. Antagonist RC-3095 is able to inhibit cancer proliferation in various cancer cells in vivo and in vitro and shows strong potential for cancer therapy. Unfortunately, a clinical trial of this antagonist failed, owing to local toxicity induced by its administration [21]. ANGII is a peptide hormone and regulates the renin-angiotensin system (RAS). Studies have shown that the upregulation of ANGII leads to the acquisition of malignancy in human cancers [22]. ANGII activates two types of ANGII receptors: AGTR1 and AGTR2. AGTR1 is overexpressed in various cancers, including breast cancer, pancreatic cancer, gastric cancer and glioblastomas [23]. ANGII is able to activate many types of signaling transduction via AGTR1 binding, including IP3/Ca^2+^ signaling, MAPKs, Src kinases and NF-κB pathways [12]. Treatment with AGTR1 antagonists, such as losartan and candesartan, show anti-tumor effects [24,25]. However, owing to ANGII’s role in the RAS, administration of the AGTR1 inhibitor might induce ventricular dysfunction and requires caution.

## 2. Nanoparticles

While the traditional methods for treating metastatic cancers, such as chemotherapy and biological therapy, are effective at killing cancerous cells, they also cause irreversible damage to normal tissues. The need for new anticancer drugs with targeted delivery has demanded a paradigm shift in cancer therapy, away from conventional methods [26]. In the meantime, recent advances in nanotechnology—for example, the development of nanomaterials and nanocarriers—have been applied to the realm of medicine, leading to the development of new drug delivery systems. Engineered nanoparticles (NPs) range in size from 1 to 100 nm. They are able to deliver cargos, such as drugs, siRNAs (short interfering RNAs) and DNAs, to the site of action without disturbing other tissues. Moreover, some of them can be delivered into the targeted cells, where they induce cell death by endocytosis. Due to the potential usage of NPs for a wide range of medical applications, a variety of nanoparticles are now being developed. The benefits of using NPs to deliver chemotherapeutic agents include improved drug solubility, stability, prolonged circulation, specific cell targeting, controlled drug release, and limited cytotoxicity, (on account of their sizes and physicochemical properties) [27,28,29]. In this review, we highlight the mechanism of targeted cancer therapy and the recent applications of nanoparticles in the treatment of GPCR-related cancers.

### 2.1. Passive Targeting and Positive Targeting

The first generation of nanomedicine in cancer therapy is based on the discovery of the enhanced permeability and retention (EPR) effect, which is used to describe the “leaky” environment of tumors. Increased cytokines, angiogenesis and other cellular factors associated with tumors, cause intratumoral vessels to lose their normal membrane lining, resulting in enhanced permeability. The pore size in tumor vessels is 100–780 nm in diameter, while the pore size in normal vessels is 5–10 nm in diameter [26]. This huge disparity in size permits the passage of certain sized drugs and their long-term accumulation. The EPR effect is exploited in cancer therapy, and allows drug circulation and accumulation, preferably in the microenvironment of the tumor. Drugs are then released by pH-dependent hydrolysis into the tumor tissue, or by endosome-mediated hydrolysis and enzymolysis [30]. Passive targeting is used by most of the clinically available anticancer nanotherapies. However, the application of passive targeting has some limitations. First, tumor cell subpopulations within the disease sites exhibit variations in phenotype and function. This is called “tumor heterogeneity” and it can lead to different cell responses towards the same drug within the target sites, and causes inconsistent outcomes [31]. Second, interstitial pressure presented in tumor sites negatively affects drug access and distribution [29]. Third, organs such as the liver and spleen have inherent endothelial fenestrations, and hence, can be accessed by drugs that do not target these organs [27]. To overcome these disadvantages, a second generation of nanomedicine, based on active targeting, is being engineered, with a focus on accurate drug delivery and enhanced efficiency.

In active targeting, the high affinity between ligands and receptors is utilized in NP delivery to achieve preferential targeting of cancer cells that express or overexpress certain receptors. The ligands are conjugated to nanocarriers and are delivered to the surface of targeted cells via molecular recognition. Similarly, antibodies can also be attached to NPs, and are transported to antigenic cell surface areas. It should be noted that in therapies used to treat solid tumors, active targeting is started after passive targeting, to allow adequate drug accumulation at the target sites [32]. It is also worth mentioning that, in the absence of specific ligands, NPs are capable of carrying out intermembrane trafficking. Palanka et al. reported that gold nanorods can attach to cell membranes via ion charges, and enter lipid membranes through transient pores formed by the generation of local heat [33].

### 2.2. Drug Design

The design of NPs depends on the purposes of application and types of action. To deliver drugs via positive targeting, NPs are often coated with biopolymers and conjugated with ligands. Drugs are either encapsulated or attached to the surface of NPs covalently in their monomeric or oligomeric forms [27,34,35]. For the drug-conjugated NPs to be delivered to the target sites, they must escape recognition and clearance by the reticuloendothelial system (RES). To achieve this, surface modulations, such as polyethylene glycol (PEG) coating or poly (HPMA) coating, are applied. The hydrophilic coating helps the NPs to escape macrophage clearance and avoid contact with plasma proteins, thus further preventing elimination by phagocytes [26].

Physicochemical properties, such as size, shape, thermal conductivity, magneticity and surface charges are often taken into consideration when designing NPs, as such properties affect how the NPs function as diagnostic tools, carriers or imaging agents. For example, smaller NPs move faster in the tumor interstitial matrix, and hence for chemotherapeutics, the size of NPs is restricted to a maximum of several nanometers [29]. The size of NPs also affects their cellular uptake rate and rate of degradation. As another example, compared to spherical NPs, nanorods are better candidates as contrast agents. This is because spherical NPs have absorption and scattering peaks in the visible region, whereas the absorption and scattering peaks of nanorods are in the near-infrared region, where the light provides broader absorption and scattering cross-sections, and deeper penetration [36]. Surface charge also plays a critical role in gene delivery. Cationic polymers are often chosen as DNA carriers. This is because positively charged polymers bind easily to the negatively charged cell membranes, making access to inside the cell possible. In addition, after enzymic actions, DNA-polymers are cleaved to DNA and polyamines. Polyamines attract hydrogen ions and chloride ions to the endosomes, which triggers endosome osmotic swelling and lysis, and prevents DNA degradation, thus allowing DNA to enter the nucleus [37].

### 2.3. Dendrimers

**Figure 1:**
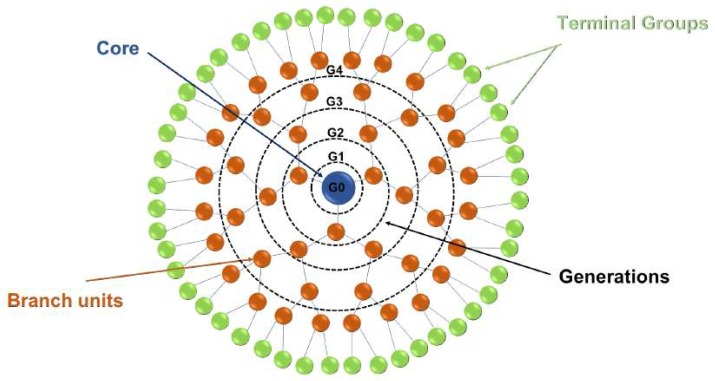
Structure of dendrimer: The blue sphere (G0) is the core of the dendrimer. Branching units are marked in orange. Starting from the core, the generations are extended and labeled as G1–G4. The outermost layer contains the dendrimer’s terminal groups (green).

Dendrimers are covalently synthesized monodisperse globular molecules embodying three parts: a core, a hyperbranched mantle and functional terminal groups [38]. The structure of the dendrimers resembles a tree, where the layers surrounding the core are connected by branching units. Each layer of dendrimer is called a generation; the core is known as G0, the first layer outside the core as G1, the second layer as G2, and so on (Figure 1). For each generation of amplification, the density of the outermost space increases. Therefore, dendrimers cannot expand unlimitedly [39]. The empty cavities in dendrimers are used to capture drugs or other small molecules. Thus, dendrimers have a high loading capacity. Furthermore, the high density of functional groups on the surface of the dendrimers makes it possible to carry multiple ligands to form multivalent ligands, which have more favorable pharmacodynamic properties than their monomeric form [40]. So far, many materials have been developed for dendrimer synthesis, such as polyamidoamine (PAMAM), polypropyleneimine (PPI), poly-l-lysine (PLL), PEG and poly-glycerol. Of these, PAMAM and PPI are more widely studied [38]. 

GPCR Ligand-Dendrimer (GLiDe) conjugates targeting adenosine receptors (ARs) are the first PAMAM dendrimers synthesized. ARs are a subfamily of Rhodopsin-like GPCRs that comprise four subtypes: A_1_, A_2A_, A_2B_ and A_3_. Each subtype has a different expression pattern in the body and different signaling pathways and performs different functions. A_1_ARs, for example, are widely located in the synaptic regions in neurons, whereas A_2A_ARs localize highly in presynaptic regions in the hippocampus. These two receptors differ from regulators of neurotransmitter release. ARs are also involved in inflammation, Parkinson’s disease and cancer [41,42]. Owing to the abundance and varied functions of the subtypes, nanocarriers with good selectivity and sensitivity (targeted to certain type(s) of ARs) are required.

Owing to improved selectivity and an affinity to receptors [43], multivalent GLiDe conjugates display enhanced pharmacokinetic and pharmacodynamic properties. The first multivalent dendrimer to activate a GPCR signaling pathway was developed by Kim et al. [44]. The dendrimer scaffold included a G3 PAMAM dendrimer as its backbone, multiple copies of A_2A_AR agonist CGS21680, and a fluorophore. Studies have shown that PAMAM–CGS21680 conjugates have the ability to inhibit ADP-induced platelet aggregation, a known function of A_2A_AR agonists. The dendrimers were found to be internalized into the platelets via receptor-mediated internalization. Following this study, a G3 PAMPAM carrier, attached to a fluorophore and multiple copies of A_2A_AR agonists (DITC-APEC), was synthesized. This conjugate showed improved conformational flexibility, ligand-receptor affinity, selectivity, (compared to PAMAM-CGS21680), and also displayed A_2A_AR agonistic behavior in the platelet aggregation assay. However, this study also revealed that, in addition to binding to A_2A_AR, the dendrimers were also bound to A_3_AR; and receptor-mediated internalization was not detected [45]. Even with these disadvantages, this pioneering research marked the discovery of GliDe applications targeting GPCRs and served as a prototype for the treatment of AR-related diseases. Other studies of GliDe conjugates targeting AR and purinergic P2Y receptors subsequently followed [46,47]. Notably, in an attempt to target heteromeric GPCR aggregates, Tosh et al. demonstrated the first example of using the same dendrimer carrier to target two different GPCRs (A_3_ and P2Y_14_) [48].

### 2.4. Quantum Dots

Originating from early efforts of the fluorescent approach to visualize receptors in the 1970’s, scientific advances have now made it possible to monitor a series of real time events of ligand–receptor interactions in living cells [49]. Among some notable advances, quantum dots (QDs) stand out due to their unparalleled fluorescence properties. QDs are also known as nano-quantum dots, semiconductor quantum dots or semiconductor nanocrystals. They are a special type of inorganic crystal with a wide excitation wavelength range, narrow and symmetrical emission wavelength, and small overlapping between excitation and emission wavelengths [49,50]. QDs possess several unique properties that make them outstanding candidates as bioimaging material. For example, their emission wavelength can be adjusted by controlling their size and composition, and the QDs of the desired wavelength can be synthesized arbitrarily [51]. In addition, QDs have considerable fluorescence intensity and stability, with no light-fading phenomenon [52]. With the rapid developments in material science and biomedical science, the use of QDs in drug screening, cell tracing, immunoassay and rapid diagnosis offers great potential [53,54,55].

Generally speaking, two main methods are used to couple QDs with biological molecules. The first method involves covalent bonding, achieved by attaching the surface of the QD with a carboxyl group, an amino group or an epoxy group. Covalent binding offers high stability and binding efficiency within a broad range of pH and ions strengths. Although the coupling procedure is relatively complicated, it can be applied to a wide range of research areas, such as antigen–antibody recognition and in vivo labeling [56]. The second coupling method involves an electrostatic adsorption method, where the charged QDs are coupled with the oppositely charged biomolecules through electrostatic interactions. This QD coupling approach is utilized in biosensors [57]. In 2005, Young and Rozengurt reported that GPCRs can be labeled by QDs in multiple cell lines. In this research, they conjugated biotinylated BBN and ANGII with streptavidin-coated QD nanocrystals with emission maxima of ~665 nm (QD655), in order to detect the interaction between GPCRs and their ligands. They successfully monitored cell-associated fluorescence of QD655-BBN in living cells that express the BBN receptor, and of QD 655-ANGII in cells expressing AGTR1. Furthermore, to investigate the performance of the QD label, they compared the strength of the signals produced by QD655-ANGII and the Cy3-labeled ANGII. QD655-ANGII exhibited much stronger luminescence and greater stability [53]. Going forward to 2015, Hennig et al. investigated the multivalent binding between QD-ANGII and its receptor. This study found that chemically modulated QD-ANGII bound to AGTR1 in a much higher affinity, compared to native ANGII in AGTR1-expressing cells, but did not bind to AGTR1-negative cells, indicating that nanoparticles bind with AGTR1 specifically and in a multivalent fashion [58]. In conclusion, QDs show great potential in cell-labeling applications, and immense potential in GPCR and cancer studies. Other than AGTR1, Fichter et al. developed a strategy using QDs to investigate the role of endosomal trafficking in serotonin receptor signaling pathways in 2010 [59]. Serotonin receptors belong to the GPCR family, which play an important role in regulating anxiety and depression-related signaling pathways. To monitor serotonin internalization and endosomal trafficking, the researchers used the biotinylated anti-HA-conjugated streptavidin QD655 to interact with the HA-tagged serotonin receptor subtype 1A (HA-5-HT1A). Both of the internalization and recycling pathways relating to 5-HT1A were successfully detected.

### 2.5. Gold Nanoparticles

The use of gold nanoparticles (AuNPs) in cancer diagnosis and treatments has been extensively investigated [60,61,62,63]. Their unique physicochemical properties make them valuable assets in a wide range of applications. First, the size of AuNPs enables them to circulate freely in the vascular system, for passive targeting. Second, AuNPs can be surface modified by the attachment of hydrocarbon chains and a variety of functional groups. This makes AuNPs an excellent multifunctional carrier. For example, AuNPs bind strongly to thiol groups due to the high affinity between sulfur and gold. Therefore, molecules containing thiol groups can be used to bridge AuNPs and a number of functional molecules, such as certain drugs, fluorescent dyes, amino acids and proteins. Thiolated biomolecules can also bind to the surface of AuNPs directly [64,65]. Additionally, AuNPs have a large surface-area-to-volume ratio, which makes it possible to attach a large number of functional groups to their surface. Furthermore, AuNPs can scatter visible and near-infrared light, and are used as imaging contrast agents. Moreover, their fluorescence-quenching ability is exploited in sensor fabrication [66,67,68]. Lastly, their surface plasmon resonance property plays versatile roles. In bioimaging, it is utilized in colorimetric sensing [69]. It is also widely used to enhance laser absorption, which leads to many therapeutic applications, such as photothermal cancer therapy, selective protein denaturation, and transmembrane drug delivery. In one study, Xiong et al. demonstrated that the use of gold nanoparticles (AuNPs) in combination with laser beams allowed the generation of water vapor nanobubbles in live cells, which caused membrane pore formation. The pores allowed the passage of macromolecules into cytoplasm without inducing noticeable cytotoxicity [70]. Other studies also described the delivery of AuNPs into cells [71] and temperature controlled delivery [72].

Jayasekara et al. were the first to synthesize AuNP carriers with non-peptide GPCR agonists [73]. In their study, several functionalized, surface-modified, hydrophilic, AuNPs-AR agonist/antagonist conjugates were evaluated for their functions and properties at the cell surface. Those AuNP conjugates showed promising potential as GPCR therapeutic targets as they were found to possess similar biological properties to their monomeric counterparts in terms of specific receptor recognition, binding affinity and receptor-induced cAMP inhibition. In 2009, Nripen et al. created novel designed gold nanorods (GNRs) and BBN conjugates, and successfully delivered them to prostate and breast cancer cells. The synthetized GNR–BBN conjugates proved to be highly stable in various solutions in vitro. Similar to BBN monomers, GNR–BBN conjugates exhibited high affinity towards cancer cells that overexpress GRPRs. Transmission electron microscope (TEM) image analysis and dark-field optical microscopy analysis have confirmed receptor-mediated endocytosis of GNR–BBN internalization [74].

### 2.6. Magnetic Nanoparticles

The design of the magnetic nanoparticle (MNP) delivery system was based on the attraction between an external magnetic field and the metal or metal oxide compounds that make up the core of MNPs. In the presence of a magnetic source, MNPs can be directed towards, and effectively “trapped” in the targeted sites. Because of their unique magnetic properties, MNPs are popularly used as contrast agents in magnetic resonance imaging (MRI), as nanocarriers, and to induce magnetic hyperthermia. As nanocarriers, external magnets are applied to provide the appropriate magnetic field gradient that allows sufficient dosage accumulation in the targeted area. Drugs or cytotoxic agents can then be released by means of passive targeting, positive targeting, or magnetically [75]. In magnetic hyperthermia therapy, MNPs are remotely controlled by an oscillating magnetic field. When arriving at the tumor sites, MNPs transform electromagnetic energy into heat and selectively kill heat-sensitive cancer cells [76].

**Figure 2:**
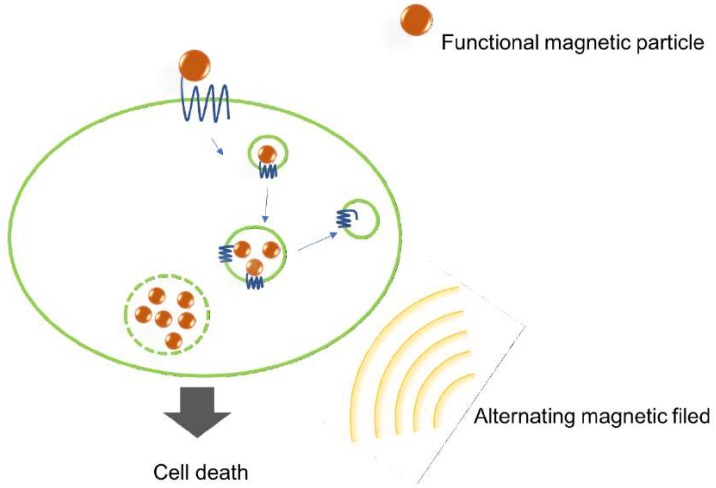
Mechanism of the cell death induced by magnetic particles. Functional magnetic nanoparticles (MNPs) recognize the surface receptor by ligand-receptor recognition. Upon binding to the receptor, MNPs are internalized and accumulated in lysosomes. When applying an external alternating magnetic field, lysosomal membranes break, which leads to cell death.

Among the classic magnetic materials, such as iron, nickel, cobalt, and their compounds, iron-based MNPs have attracted great attention, owing to their size-dependent magnetic properties and excellent biocompatibility, compared to other magnetic materials [76,77]. Superparamagnetic iron oxide NPs (SPIONs), for example, have been intensively studied and are used in a variety of biological applications. Richard et al. engineered a bimodal imaging tracer using a PEGylated SPION nanoplatform. It was chemically modified and bound to a fluorophore and an antibody, against an endothelin receptor (ETA)—a GPCR that overexpressed in a variety of solid tumors. Not only did the SPION conjugates demonstrate effective ETA targeting, showcasing the antibody’s resuming functionality after conjugation, but they also manifested low toxicity and high stability under physiological conditions. Their excellence as an MRI contrast agent and imaging tracer has also been demonstrated by in vivo experiments in mice [32]. Sanchez et al. investigated cell death induced by iron oxide nanoparticles (IONPs) targeting cholecystokinin-2 receptor (CCK2R). CCK2R is overexpressed in thyroid carcinomas, small-cell lung cancers and gastrointestinal stromal tumors. Both of its ligands, (gastrin and CCK), can activate CCK2R. A synthetic form of gastrin, termed MG, was engineered to have a pharmacophore of gastrin, a CCK C-terminal sequence (used to investigate CCK2R internalization), and an exchanged amino acid sequence (aimed at preventing the accumulation of gastrin in kidney). MG was then conjugated with IONP and a fluorescent label DY647. The fate of the conjugates was subsequently revealed. In cancer cells, they specifically bound to CCK2Rs, and underwent receptor-dependent endocytosis before entering lysosomes. When subjected to an external magnetic field, the internalized NPs caused lysosome leaking, which led to cell death [78]. The mechanism of MNP-induced cell death is illustrated in Figure 2.

### 2.7. Others

In addition to the examples mentioned above, other materials that target GPCR-related cancer have been investigated. For example, Fan et al. synthesized unmodified NPs, conjugated with follicle-stimulating hormone polypeptide (FSHP) and anticancer drug PTX, to treat lymphatic metastasis in ovarian cancer therapy. The conjugates were administered to mice injected with ovarian cancer cells. Compared to control groups (NP-PTX and FSHP-NP), FSHP-NP-PTX showed the highest PTX concentration in lymph nodes, the greatest inhibitory effect in cell proliferation, and increased mice survival time. Furthermore, PTX toxicity seemed to be reduced when the PTX was conjugated with NPs [79]. As another example, in an attempt to target GRPRs in prostate cancer, Zhang et al. developed an elastin-like polypeptide (ELP) micellar carrier platform fused with GRP. Specific receptor targeting was achieved, as demonstrated by intracellular calcium release. GPR binding to membrane and NP internalization was also monitored [80]. Table 1 lists some of the more recently discovered NPs and their application in GPCR-related studies. 

## 3. Conclusions

The GPCR gene family, the largest group of GPCRs, is the molecular target that accounts for almost 30% of FDA-approved drugs [89]. The demand for pharmaceuticals that target GPCRs with good selectivity and enhanced pharmacokinetic qualities, has driven the development of alternative therapies. In GPCR-related cancer treatments and diagnosis, NPs have provided a new mode. NPs have versatile functions. They are used as imaging agents, contrast agents and therapeutic carriers, as well as to induce cell death via endocytosis. As an imaging agent, NPs are able to detect cancer at very early stages, as they are sensitive towards receptors that are only expressed in cancer cells. They have the potential to distinguish cancer cells before the cells migrate and become solid tumors. Early cancer diagnosis is important to prognosis and the outcome of treatment. In addition, NPs are of great value in detecting cancer progression and migration and have proved to be valuable agents in cancer research. In therapeutics, NPs are not only engaged in targeted delivery, but also able to form multivalent ligands with increased receptor binding affinity and avidity, which leads to enhanced pharmacokinetic and pharmacodynamic properties. The applications of NPs in cancer research are expanding with advances in technology and therapeutic strategies.

GPCR signaling regulatory pathways are intricate and complex, but more are yet to be discovered. For this reason, nanoparticle ligand-based complexes represent an alternative approach to the study of GPCRs. 

## Figures and Tables

**Table 1:** Recent applications of NPs targeting GPCR.

Nanoparticles	Ligand/Antibody	GPCR	Function	Reference
Dendrimer	MRS2500 (antagonist)	P2Y(1)	Affinity to receptor (K_i_ 23 nM); Inhibiting ADP-promoted human platelet aggregation; Antithrombotic drug	[46]
	XAC (antagonist)	Adenosine receptor (A_2A_AR)	Affinity to receptor (K_i_ 3.7 nM); Treating Parkinson’s disease and asthma	[81]
	CGS21680 (agonist)	Adenosine receptor (A_2A_AR)	Affinity to receptor (K_i_ 12 ± 6 nM); Activating adenylate cyclase and accumulating cAMP; Inhibiting ADP-promoted human platelet aggregation; Using short PEG chains in nanocarriers targeting ligand-receptor interactions	[82]
	UDPGA-A3AR 3a-G4 PAMAM (agonist)	Both Adenosine receptor (A_3A_R) and P2Y14	Affinity to receptor (K_i_ 39.5 nM to A_3A_R); Inhibiting cAMP cumulation; Antithrombotic drug	[46]
	DITC-APEC (agonist)	Adenosine receptor (A_2A_AR)	Affinity to receptor (K_i_ 70 ± 3 nM); Inhibiting ADP-promoted human platelet aggregation; Antithrombotic drug	[45]
Quantum dots (QDs)	α-melanocyte-simulating hormone NDP and MT-II	Human melanocortin receptor (hMCR)	Specific marking of surface receptors; Single molecule imaging of GPCR; Multiplexing; Investigating GPCR localization and trafficking in live cells and in single molecule studies	[83]
	Deltorphin-II	Human δ-opioid receptor (hDOR)		
	ANGII	ANGIIReceptor	Monitoring ligand-receptor binding in live cells; Monitoring GPCR internalization; Detecting and labeling several GPCRs in living cells	[53]
	BBN	BBN receptor		
	HaloTag ligand	Cyclic AMP receptor 1 (cAR1)	Single molecule imaging of GPCR; Using multi-colored, single molecule imaging to study membrane protein dynamics	[56]
	Antibody against flag tag (HA)	Serotonin receptor subtype 1A (5-HT_1A_)	Monitoring internalization and endosomal trafficking; Identifying two distinct GPCR recycling pathways	[59]
	ANGII	AGTR1	Higher affinity to receptor than native ANGII; Multivalent ANGII-targeted NP-AGTR1 binding; Targeting tissues that overexpress AGTR1	[58]
	Adenosine	Adenosine receptor (A_2A_AR)	Studying the mobility of the receptor in neurons	[84]
	Antibody against flag tag (GFP)	Serotonin receptor subtype 1B (5-HT_1B_)	Monitoring receptor trafficking in rat hippocampal neurons; Suggesting alternative mechanism of mood regulation via GPCR	[85]
	Antibody against flag tag (HA)	κ-opioid receptors (κ-ORs)	Displaying receptor affinity and specificity; Monitoring receptor trafficking; Detecting two receptors in one cell; Application in drug screening	[86]
	Antibody against flag tag (GFP)	Adenosine receptor (A_2A_AR)		
	Antibody against flag tag (GFP)	Endothelin A receptor (ET(A)R)	Monitoring receptor intracellular translocation; Detecting intracellular targets (when conjugated with a cell penetrating agent)	[87]
	Antibody against flag tag (HA)	Serotonin receptor (5-HT_1A_)	Single molecule imaging to monitor receptor trafficking; Understanding the mechanism of therapies targeting GPCR	[59]
Gold nanoparticle (AuNP)	Several AR agonists and antagonists	Adenosine receptor (AR)	Affinity to receptor (K_i_ 37 nM to A_3A_AR); Cancer diagnosing and treatment	[73]
	BBN	Gastrin-releasing peptide receptor (GRPR)	Monitoring receptor internalization; Cancer therapy and molecular imaging in vivo	[74]
Magnetic nanoparticle (MNP)	Anti-ET_A_ antibody	Endothelin receptor (ET_A_)	Efficient receptor targeting; Bimodal contrast agent and imaging agent; Targeting tumor pathologies; Early Cancer diagnosing	[32]
	MG	Cholecystokinin-2 receptor (CCK2R)	Inducing cell death in cancer	[59]
Elastin-like polypeptide (ELP) micelles	GRP	Gastrin-releasing peptide receptor (GRPR)	Specific receptor targeting; Delivering cytotoxic drugs to cancer	[80]
Unmodified NPs	FSH	Follicle-Stimulating hormone receptor (FSHR)	Successfully delivering drug to lymph nodes; Reducing metastasis; Ovarian cancer therapy	[79]
Nanorubies	Antibody against flag tag (HA)	μ-opioid receptor	Imaging functionality; Achieving real-time single molecule imaging on biological samples	[88]

## Abbreviations

ANGII	Angiotensin II
AR	Adenosine Receptor
AuNP	Gold Nanoparticle
CCK2R	Cholecystokinin-2 Receptor
CXCR4	Chemokine Receptor 4
EGFR	Epidermal Growth Factor Receptor
ELP	Elastin-Like Polypeptide
EPR	Enhanced Permeation and Retention Effect
ETA	Endothelin Receptor
GLiDe	GPCR Ligand-Dendrimer
GNR	Gold Nanorod
GPCR	G Protein-Coupled Receptor
GRK	G protein-Coupled Receptor Kinase
GRPR	Gastrin-Releasing Peptide Receptor
GTP	Guanosine Triphosphate
5-HT1A	Serotonin Receptor Subtype 1A
IONP	Iron Oxide Nanoparticle
LPAR	Lysophosphatidic Acid Receptor
PDGFR	Platelet-Derived Growth Factor Receptor
RTK	Receptor Tyrosine Kinases
